# Evidence, theory and context - using intervention mapping to develop a school-based intervention to prevent obesity in children

**DOI:** 10.1186/1479-5868-8-73

**Published:** 2011-07-13

**Authors:** Jennifer J Lloyd, Stuart Logan, Colin J Greaves, Katrina M Wyatt

**Affiliations:** 1 Institute for Health Service Research, Peninsula College of Medicine and Dentistry, University of Exeter, Exeter, UK

## Abstract

**Background:**

Only limited data are available on the development and feasibility piloting of school-based interventions to prevent and reduce obesity in children. Clear documentation of the rationale, process of development and content of such interventions is essential to enable other researchers to understand why interventions succeed or fail.

**Methods:**

This paper describes the development of the Healthy Lifestyles Programme (HeLP), a school-based intervention to prevent obesity in children, through the first 4 steps of the Intervention Mapping protocol (IM). The intervention focuses on the following health behaviours, i) reduction of the consumption of sweetened fizzy drinks, ii) increase in the proportion of healthy snacks consumed and iii) reduction of TV viewing and other screen-based activities, within the context of a wider attempt to improve diet and increase physical activity.

**Results:**

Two phases of pilot work demonstrated that the intervention was acceptable and feasible for schools, children and their families and suggested areas for further refinement. Feedback from the first pilot phase suggested that the 9-10 year olds were both receptive to the messages and more able and willing to translate them into possible behaviour changes than older or younger children and engaged their families to the greatest extent. Performance objectives were mapped onto 3 three broad domains of behaviour change objectives - establish motivation, take action and stay motivated - in order to create an intervention that supports and enables behaviour change. Activities include whole school assemblies, parents evenings, sport/dance workshops, classroom based education lessons, interactive drama workshops and goal setting and runs over three school terms.

**Conclusion:**

The Intervention Mapping protocol was a useful tool in developing a feasible, theory based intervention aimed at motivating children and their families to make small sustainable changes to their eating and activity behaviours. Although the process was time consuming, this systematic approach ensures that the behaviour change techniques and delivery methods link directly to the Programme's performance objectives and their associated determinants. This in turn provides a clear framework for process analysis and increases the potential of the intervention to realise the desired outcome of preventing and reducing obesity in children.

## Background

Over a very short timescale there has been a substantial increase in the proportion of children in the UK who are overweight [1] The Health Survey for England (2008) reported that 19% of girls and 18% of boys aged 11-15 were obese and 34% of girls and 33% of boys were overweight [1]. The National Child Measurement Programme reported that by age 10-11 years (Year 6) one in three children were either overweight or obese [2]. Being overweight in childhood is associated with adverse consequences including metabolic abnormalities, increased risk of Type II diabetes, and musculo-skeletal and psychological problems [3]. Over 50% of obese children become obese adults [4] with significant health consequences [5].

Unfortunately there is currently little evidence that existing, school-based intervention programmes are effective in preventing or reducing obesity in children. In addition, most intervention programmes have not reported on their rationale, development, exact content, or method of implementation which further hampers our understanding about what works and why. In tackling childhood obesity, securing scientific information on what constitutes a healthy diet and an active lifestyle is only the first step. The second step, requiring an equally scientific approach, is to find methods of achieving behaviour change. The determinants of behaviours linked to obesity are complex and inevitably changing these behaviours is difficult and interventions are likely to be complex and multi-faceted. The 2008 MRC Framework for developing and evaluating complex interventions recommends that the mechanisms by which interventions work need to be made explicit during development [6] and such interventions need to be comprehensively described if they are to be replicable by others. This is important as it provides a basis for checking intervention fidelity, a necessary pre-requisite to understand efficacy. It also provides a basis for process analysis (relating mechanisms of change to outcomes) which can shed light on why complex interventions succeed or fail and how they can potentially be optimised.

Schools have the potential to play a critical role in the prevention of overweight and obesity. With their existing organisational, social and communication structures they provide opportunities for regular health education and for a health enhancing environment. They also enable the researcher to engage children and families across the social spectrum. In England, children attend a primary or junior school up to the age of 11, where they usually have one class teacher who teaches all subjects. This allows for joined up cross-curriculum activities and facilitates communication making both intervention and research in this setting particularly attractive.

**Figure 1:**
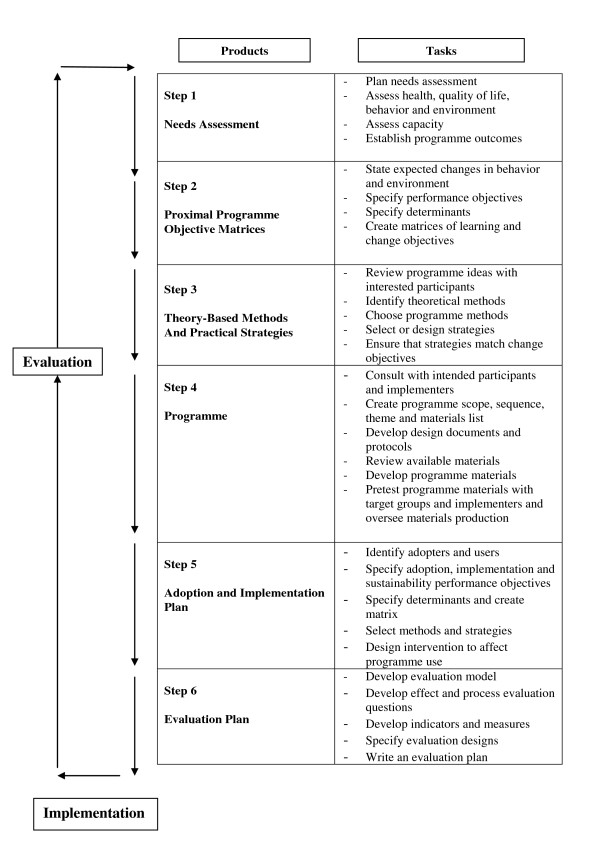
**The Intervention Mapping process**.

In this paper we describe the application of a systematic process, Intervention Mapping (IM) (see Figure 1) [7] to plan a school-based obesity prevention intervention.

## Methods

### Intervention Mapping

The six main steps of IM (Figure 1) are: i) needs assessment; ii) detailed mapping of programme objectives and their behavioural and environmental determinants; iii) selecting techniques and strategies to modify the determinants of behaviour and the environment; iv) producing intervention components and materials; v) planning for adoption, implementation and sustainability; and vi) creating evaluation plans and instruments. IM uses behavioural theory and research evidence to develop specific learning and change objectives for the target population and to identify the personal and external determinants of these objectives. Theory and other considerations (e.g. stakeholder opinions, feasibility data) also guide the choice of intervention methods and strategies to achieve these objectives. We used a variety of methods to gather the appropriate information to enable us to produce a feasible and acceptable intervention that has the potential to change behaviours at a school, child and family level. These included literature reviews, discussions with stakeholders (teachers, head teachers, education advisors, local public health leads in physical activity and obesity) and experts in behavioural science and obesity research. We also carried out focus groups with children and interviews with parents and teachers during early pilot work to inform our selection of intervention techniques and strategies and to ensure that these remained feasible to deliver within normal school activities.

The following sections provide a summary of the first 4 steps of the IM process used to produce the HeLP intervention. Steps 5 and 6 involve programme implementation, adoption, monitoring and evaluation and are not presented here. While the steps are described in linear fashion they are, in fact, iterative. For example, defining a more specific behaviour change objective (e.g. parents need to buy and provide healthier snacks) might lead to the consideration of additional behavioural determinants (those which affect parental shopping behaviours as well as those which affect the child's eating behaviour).

### Step 1: Needs Assessment

The IM process begins with a needs assessment of the health problem, which includes identification of the problem behaviours (and to some extent their determinants) and of desired programme outcomes as well as the environmental conditions associated with the problem.

#### Reviewing the evidence base

The starting point was to review the literature to identify (i) risk factors for childhood obesity and children's current eating/drinking and physical activity behaviours (ii) the determinants of these behaviours and (iii) apparently successful and unsuccessful components of previous school-based interventions to prevent and reduce obesity.

##### (i)Possible risk factors for obesity

Obesity results from an imbalance between consumption and expenditure of energy. Controlled experimental and epidemiological studies suggest a number of dietary risk factors associated with increased energy intake in children and adults. These included, diets with a high energy density [8] usually characterised by foods high in fat and low in fibre, including fast food [9,10] and large habitual portion sizes [11]. Experimental studies also report that liquid calories have lower satiating properties than solid food [12] and epidemiological studies report an increased risk of weight gain or obesity in consumers of sugar-rich drinks. A single carbonated drink per day can add 10% to a child's energy intake [12]. According to the National Diet and Nutrition Survey (2008/9), in the UK children's intake of non milk extrinsic sugars (NMES) provides 15% of food energy [13], compared to a recommendation of not more than 11% [14]. Carbonated soft drinks are a major source of NMES providing 19% of NMES intake in children aged 4-10 and over one-third in children aged 11-18 [13].

Reduced energy expenditure has also been associated with weight gain [15] and numerous studies in adults and children reported an association between lower weight gain and higher levels of physical activity [16]. Stratton et al reported a decrease in the levels of cardiovascular fitness in 9-11 year olds in England between 1997 and 2003 while the prevalence of obesity increased over the same time period [17]. Children's TV viewing time and time spent playing electronic games has been associated with overweight and obesity [18-20], total calorific intake [21] and the consumption of snack foods [22]. Longitudinal data from the Avon Longitudinal Study of Parents and Children (ALSPAC), found strong associations between children's fat mass at age 14 and their physical activity at age 12 [23]. We also know that today's children are spending more time in front of the television or computer screen than in previous generations - an average of two and a half hours of TV and 1 hour and 50 minutes online a day [24]. (i.e. nearly 4 1/2 hours a day of screen time). An attempt to encourage children to replace screen-based sedentary behaviours with more active pursuits is clearly an appropriate aim in preventing obesity in children and promoting a healthy lifestyle.

##### (ii)Determinants of behaviours

A variety of family and social determinants affecting children's eating and activity behaviours have been identified. For eating, these include food preferences, food availability and accessibility, modeling (copying the behaviour of others), mealtime structure (social context of meals, the role of TV during mealtimes, eating out, portion size, school meals, snacking habits), feeding styles (the caregivers approach to maintain or modify children's behaviours with respect to eating) and socio-economic and cultural factors (e.g. family time constraints, education, income, ethnicity and culture) [25]. In terms of children's physical activity, parental support (e.g. transporting the child, observing the activity, encouraging the child, providing equipment, participating with the child and reinforcing physical activity behaviours) has been identified as a key determinant both directly and indirectly through its positive association with self efficacy perceptions [26]. Griew et al recently reported that children's school time physical activity varied according to the primary school they attended even after accounting for individual demographic and the school compositional factors with a 'school effect' explaining 14.5% of the variation in pupils' school-time physical activity [27]. However, it is less clear that school based activities have a substantial effect on total, as opposed to school time, activity. In a study of 3 schools from one area, with different sporting facilities and opportunity for physical activity in the curriculum, Mallam et al (2003) reported large differences in school time activity levels but virtually no differences in the total activity of the children [28].

This research suggests that while it appears that schools have the potential to create a positive physical activity culture that can influence whether children engage in physical activity it will be crucial in intervention studies to assess whether any effects translate in to changes in total as opposed to only school time activity.

Drawing on the social ecological approach [29] we began from the theoretical perspective that, while both eating and activity behaviours in children are partly determined by choices made by the children, they are highly dependent both on direct intervention by parents (e.g. the food provided, opportunities for physical activity) and by patterns of behaviour within the family, within the school and within peer groups. As children get older the relative importance of self directed, as opposed to family directed, behaviours increases and these behaviours are influenced by wider social factors which include the school environment and peer group norms. Therefore any intervention we designed needed to affect behaviour through influencing the children, their families and the school environment. There is some evidence from previous studies of interventions in children that the use of drama/theatre can be an effective tool to engage children, increase knowledge and change behaviours [30-33]. For example, in an obesity prevention programme aimed at low income children and their parents, an after school theatre-based intervention was shown to motivate and engage both parents and children and increase awareness of the need for making changes. However, the authors did conclude that theatre alone is not enough to lead to behavioural change and that the next step should be to incorporate this delivery method into more comprehensive programmes with both educational and environmental components [31]. Two small studies in primary schools in the UK based on drama/the arts reported increases in vegetable, salad and fruit juice consumption [32,33]. Although both these studies had serious methodological weaknesses, the use of drama to engage children to change specific behaviours looked promising and was explored at length with experts from drama and education as a possible implementation strategy in step 3 of the intervention mapping process.

We were mindful that there were other key drivers including intrinsic factors such as genes and the wider social environment but these are less modifiable and so were not considered as potential points of intervention.

##### (iii) School-based interventions

The most recent systematic review (2009) of controlled trials of school-based interventions identified 38 studies; 3 dietary intervention only, 15 physical activity only and 20 combined diet and physical activity [34]. The authors concluded that there was insufficient evidence to determine the effectiveness of dietary interventions alone, but suggested that interventions which increase activity and reduce sedentary behaviour may help children to maintain a healthy weight, although results were short-term and inconsistent. Results for combined diet and activity were also inconsistent, although there was a suggestion that the combined approach might be more effective in preventing children becoming overweight in the long term. Social Cognitive Theory (SCT), which proposes that a dynamic interaction exists between personal, behavioural and environmental factors, provides a basis for many of these programmes, particularly the constructs of self efficacy, behavioural capability (knowledge and skills to perform a behaviour), outcome expectations, self regulation and reinforcement [35]. Environmental conditions of eating behaviour such as school lunch provision and parental/home environment were often targeted [36,37]. A review of reviews of effective elements of school health promotion across behavioural domains (substance abuse, sexual behaviour and nutrition) found that five elements from the highest quality reviews were found to be effective for all three domains using two types of analysis. These were use of theory; addressing social influences (especially social norms); addressing cognitive behavioural skills; training of facilitators and multiple components. Using one type of analysis only, another two elements were identified: parental involvement and a large number of sessions [38].

The authors concluded that the 5 elements identified should be primary candidates to include in programmes targeting these behaviours.

#### Stakeholder consultation

A second approach to needs assessment is to collect information to enable a deeper understanding of the context or community in which the intervention is to be delivered [7]. The next step in our needs assessment was therefore to run a workshop with practitioners, policy makers and researchers from education, child health, sports science, the local PCT and the local healthy schools team. In the workshop we addressed the nature of the problem and the findings of our literature review, seeking ideas about possible behavioural objectives for schools, children and their families and what the desired outcomes of the programme should be.

This workshop resulted in agreement about four key principles which it was suggested should guide our intervention design. Firstly, that a public health approach should be adopted including all children rather than targeting the overweight. The adverse health consequences of obesity are not limited to those at the extreme end of the BMI distribution and, although most children remain lean, many will gain weight as adults. In addition, separating children within a class for special intervention risks stigmatising them. Secondly, the intervention needed to engage parents and offer them strategies through which they could directly (through parenting) or indirectly (through the creation of supportive environments) foster the development of healthy eating and activity behaviours among their children/family. Thirdly, in order to provide an intervention that was not only feasible and acceptable to schools, but had potential for long term sustainability, the intervention should dovetail with healthy lifestyle initiatives already present in schools and aim to meet National Curriculum requirements for the age group targeted, something previously recommended by Doak et al (2006) in a review of interventions and programmes to prevent obesity in children [39]. Finally, the methods chosen to deliver the intervention to children and parents not only needed to engage, motivate and inspire but should also be realistically deliverable by teachers and relevant external groups operating within a school setting.

#### Outputs

Based on the above needs assessment process we decided to develop an intervention which aimed to support children to achieve small sustainable changes across childrens' patterns of diet and physical activity but with a focus on three key behavioural objectives:

1. to reduce the consumption of sweetened fizzy drinks

2. to increase the proportion of healthy snacks consumed and

3. to reduce TV viewing and other screen based activities.

### Step 2: Detailed mapping of programme objectives

Step 2 provides the foundation for intervention development by specifying in detail who and what will change as a result of the programme. The products of step 2 are *proximal programme objectives *or PPOs. These are statements of demonstrable behaviours (in the target group) or changes in the environment that need to occur in order affect the determinants of the overall behavioural objectives that have been identified in step 1 (and further refined in step 2). To define PPOs, we first defined key behavioural objectives (see above) and broke these down into smaller steps (performance objectives) and then identified the determinants of each performance objective. Then we specified 'proximal programme objectives' (i.e. the most immediate targets of intervention - what needs to be learnt or changed in order to modify behavioural determinants and consequently the key behavioural objectives).

As the aim of our intervention was to develop a school-based intervention which was delivered to children but was able to influence parents and the school as well, activities needed to include parents/families, teachers and the senior management team (SMT). Further, more specific behavioural objectives, called *performance objectives *(POs) were developed for each group (children, parents/family, teachers, SMT). These constituted individual behaviours, motivations, abilities and environmental opportunities in the home and within the school for each group in order for the three key behavioural objectives to be achieved. The performance objectives developed for the parents/family, teachers and the SMT were focused on engaging the school and the children's families in order to create the necessary conditions to enable children make sustainable changes to their eating and activity behaviours. For example, at the outset, a PO for the SMT was for them to 'buy into' the Programme and believe it would benefit the school and the children and would dovetail with the existing year 5 curriculum and school initiatives already in operation. For the purposes of this paper we will confine our examples to the performance objectives related to the child, however, a detailed intervention specification supporting this paper is available to view (See Additional file 1) which shows the POs, determinants (change targets), BCTs and methods of delivery for all the target groups.

#### a) Defining overall behavioural objectives

The creation of a behavioural objective requires breaking down the desired outcome, in this case, preventing obesity, into component parts that influence or are required to achieve the desired outcome. The three key target behaviours, reducing consumption of sweetened fizzy drinks, increasing the proportion of healthy snacks consumed and reducing TV viewing and other screen-based activities were expanded into a set of sub-component behaviours (performance objectives, POs). These performance objectives clarified the exact behavioural performances expected from children, parents and teachers in order to meet these key objectives and referred to individual level behaviours, motivations, abilities as well as to environmental opportunities for such behaviours at the home and school level. As involvement of parents was vital in achieving the three key target behaviours, we knew we needed children to clearly communicate the messages to their parents and engage them in supporting their goals. This was originally construed as a PPO related to the determinants of social support, modelling and reinforcement but was promoted to a PO so that the intervention could explicitly focus on strategies to promote this dialogue between the child and their family. The iterative process of identifying performance objectives was added to over time as the mapping process identified additional issues. For example the concept of enabling children to recognize and resist temptation for unhealthy snacks was originally a PPO (which aims to address the determinant of 'urges for unhealthy foodstuff' as related to the objective of 'reducing unhealthy snacks') which we also promoted to a performance objective to allow a more detailed analysis of this key issue. Although this process was time consuming, it was useful in creating a more focused and considered intervention.

#### b) Identification of Determinants

In order to specify our 'change targets' i.e. those potentially modifiable determinants of obesity related behaviours we i) reviewed the determinants of children's eating and physical activity behaviours reported by experimental and epidemiological studies and components of previous school-based interventions to prevent and reduce obesity; ii) sought expert opinion from an advisory panel of researchers in the field and behavioural scientists; and iii) made reference to theories of behaviour and/or behaviour change. The determinants were categorised as personal (factors within the individual under their direct control) or external (factors outside of the individual that can directly influence the health behavior or environmental conditions). The final list of determinants to be targeted is provided in Table 1. These were selected based on their links to theoretical models of behavior change which have formed a basis for previous school-based interventions and their potential to be modified within a school setting.

**Table 1:** Examples of determinants of eating and physical activity behaviour in children targeted by the Healthy Lifestyles Programme

Personal Determinant	External Determinants
Knowledge and skills to perform tasks required by the intervention (e.g. communicating with parents, select healthy snacks/drinks)	Norms

Food preferences and perceived enjoyment	Modelling by parents

Food cravings (urges for unhealthy foods)	Modelling by peers

Activity preferences and perceived enjoyment (sedentary activities vs more active pursuits)	Availability and accessibility of healthy and unhealthy foods in and outside the home and in the school environment

Perceived familiarity of foods/physical activities	Availability and accessibility of physical activity opportunities in school and during parental care

Perceived norms regarding choice of food/leisure activities in family and peer group	Family support (emotional, instrumental and informational)

Self efficacy regarding selection of food/physical activity	Reinforcement from parents, teachers and peers

Self awareness regarding diet and physical activity and screen-based sedentary behaviours	

Attitude to the Programme (intention to make changes)	

Perceived importance of eating healthily and exercising (pros and cons of making a change)	

A focus on delivering the Programme in such a way that children enjoyed the activities and were motivated to participate was also seen as a key determinant for a number of POs, as affective responses are linked to both physical activity and eating behaviours. It is likely that children will be motivated and enjoy activities if they have positive attitudes towards the behaviour [40], feel competent to make changes [41], perceive significant others to be motivated and perceive they have some control over outcomes [42]. The main determinants or 'change targets' for the HeLP Programme therefore, were (i) knowledge and skills (ii) self efficacy, (iii) self awareness, (iv) taste, familiarity and preference, (v) perceived norms (vi) support, modelling and reinforcement from family members and (vii) access and availability of opportunity. Having selected our change targets or determinants the next step was to identify the specific behaviours necessary to modify them.

#### c) Define proximal program objectives

The final part of this step is to define the *proximal programme objectives *(PPOs) by mapping performance objectives (row headings in tables 2, 3 and 4) against determinants (column headings in table 2, 3 and 4) in a table to form a matrix. In the tables, cells created from personal determinants record what the target group should do and/or know and cells created from external determinants record what should change in the environment in order for there to be a positive impact on each determinant so that the performance objective can be achieved. These end statements are the PPOs. For example, for children to communicate healthy lifestyle messages to parents and seek their help and support, change in three personal and two external determinants are required (see Table 2). From a personal perspective, the child needs specific knowledge and skills to communicate the messages to their parents and seek their help and support (taught throughout the intervention using a variety of methods) and perceive that their peers are talking about the project and also seeking their parents support. Practising communication through role play and engaging parents using homework tasks, drama productions and school assemblies may increase self efficacy in communicating messages to parents and making suggestions for support. From an external perspective, the child requires support and reinforcement from parents, teachers and peers. This increased communication with parents/family needs to increase family awareness of healthy lifestyles and in turn lead to the family increasing availability and accessibility of healthy snacks and active pursuits at home.

**Table 2:** Matrix of performance objectives and determinants for 'Establish Motivation'

		Personal Determinants				External Determinants	
**Performance Objectives**	***Knowledge/*** ***Skills***	***Self-efficacy***	***Self-awareness***	***Taste Familiarity Preference***	***Perceived norms***	***Family support, Modelling Reinforcement***	***Availability*** ***Accessibility***

*A Communicate healthy lifestyle messages to parents and seek their help and support	^1^Understands messages and energy balance concept ^2^Practices skills to communicate with parents ^3^Understands how parents can support a healthy lifestyle ^4^Practices skills to seek parental support	^5^Shows confidence knowledge of healthy lifestyle ^6 ^Shows confidence to talk to parents ^7^Shows confidence and knowledge of family strategies to support a healthy lifestyle ^8^Shows confidence to seek parental support			^9^Perceives other pupils are talking about the project ^10^Perceives others are seeking parental support	^11^Receives social reinforcement from parents/family for interest in healthy lifestyles ^12^Receives reinforcement from parents/family for suggested support strategies	^13^Increases in availability of healthy snacks/drinks and active pursuits

**B** **Select and try healthy alternatives to unhealthy snacks and drinks at home and at school**	^14^Identifies healthy alternatives to unhealthy snacks and drinks ^15^Practices skills to ask for healthy alternatives in different settings ^16^Taste healthy alternatives to unhealthy snacks and drinks	^17^shows confidence to select healthy snacks and drinks ^18^shows confidence to try new snacks and drinks		^19^Is familiar with and chooses healthy snacks and drinks	^20^Perceives family, peers, teacher expecting them to select healthy alternatives	^21^Receives reinforcement from family, peers and teachers	^22^Increases in availability and accessibility of healthy snacks and drinks at home

**C** **Select feasible active alternatives to sedentary activities**	^23^Identifies active alternatives to sedentary leisure pursuits ^24^Attends activity workshops Participates in active games	^25^Shows confidence and enthusiasm		^26^Is familiar with range of active alternatives to sedentary pursuits	^27^Perceives family expecting active choices	^28^Receives reinforcement from family, peers, teachers	^29^Increases in active leisure opportunities at home

D *Understand and resist temptation	^30^Identifies general barriers to being healthy ^31^Understands marketing strategies used to tempt children ^32^Practices skills to resist temptations	^33^Shows confidence to resist temptation	^34^Records what tempts them into eating unhealthy snacks and drinks and being sedentary		^35^Perceives peers and family are resisting temptation	^36^Sees parents, family and peers resist temptation	^37^Decreases in temptations in the home

* POs originally construed as PPOs which have been promoted to a higher level

**Table 3:** Matrix of performance objectives and determinants for 'Take Action'

		Personal Determinants				External Determinants	
**Performance Objectives**	***Knowledge/*** ***Skills***	***Self-efficacy***	***Self-awareness***	***Taste Familiarity Preference***	***Perceived norms***	***Family support, Modelling Reinforcement***	***Availability*** ***Accessibility***

**E** **Reflect own snacking and leisure choices**	^38^Identifies unhealthy snacks in diet and sedentary leisure choices ^39^Compares to guideline	^40^Shows confidence in ability to assess own behaviour	^41^Completes 2 day food record ^42^Completes 24 hour activity record			^43^Receives reinforcement from parents and teachers ^44^Sees peers evaluate snacking and activity choices	

**F** **Set goals and make changes**	^45^Knows role of goal setting in helping to change behaviours ^46^Knows goals need to be SMART ^47^Writes 3 SMART goals ^48^Knows range of strategies to help achieve goals ^49^Identifies personal strategies to help achieve goals	^50^Shows confidence in ability to make small changes			^51^Perceives peers are making changes	^52^Receives reinforcement from parents and family	^53^Increases in the availability and accessibility of healthy snacks and drinks at home ^54^Increases in active leisure opportunities at home

**Table 4:** Matrix of performance objectives and determinants for 'Stay Motivated'

		Personal Determinants				External Determinants	
**Performance Objectives**	***Knowledge/*** ***Skills***	***Self-efficacy***	***Self-awareness***	***Taste Familiarity Preference***	***Perceived norms***	***Family support, Modelling Reinforcement***	***Availability*** ***Accessibility***

**G Monitor goals**	^55^Produces a personal monitoring chart ^56^Knows 80/20 message	^57^Shows confidence in monitoring goals	^58^Completes personal monitoring chart		^59^Perceives peers are monitoring goals	^60^Receives reinforcement from teachers and parents for monitoring goals	

**H** **Assess barriers to goal achievement**	^61^Knows how their environment affects their choices ^62^Knows how personal temptations have affected achieving goals ^63^Plans new strategies to overcome barriers	^64^Shows confidence to overcome barriers experienced	^65^Records barriers and strategies		^66^Perceives peers planning strategies	^67^Receives reinforcement from teachers and parents	^68^Increases in availability and access to healthy snacks and drinks at home

**I** **Adapt goals**	^69^Knows if goals are SMART ^70^Knows how to adapt goals	^71^Shows confidence to adapt goals based on experience				^72^Receives social reinforcement from parents for being motivated	^73^Increases in active leisure opportunities and healthy snacks and drinks at home

SMART goals - goals that are Specific, Measurable, Achievable, Realistic and Time-based

The end point of step 2 in the intervention mapping process, i.e. defining proximal programme objectives, is an iterative process and we moved back and forth between the tasks of defining POs and their associated determinants from the ones targeted in the HeLP Programme (see Table 1) and the creation of statements of demonstrable behaviours. e.g. 'practices skills to seek parental support' that would modify a particular determinant and thus help achieve the performance objective. This process produced an overwhelming amount of information which we had to condense in order to develop a feasible and acceptable intervention within the school setting.

During the process of creating the matrix, in order to guide the sequential order in which behaviour change techniques were delivered in our intervention, we decided to map performance objectives onto a process model of behaviour change. The Health Action Process Model (HAPA) [42] was selected as a 'starting point' as it is consistent with the theoretical models of behaviour change mentioned earlier and suggests that behaviour change occurs through a sequence of adoption, initiation and maintenance processes. This phased model implies a clear order of distinct actions which is easily understood and is compatible with a sequential application of techniques spread across the curriculum of a school year. By taking these phases into account, performance objectives and their associated PPOs were mapped onto three processes of behaviour change; *Establish motivation *(develop confidence and skills, make decisions); *Take action *(create an action plan and implement it); *Stay motivated *(monitor progress, assess and adapt goals).

Tables 2, 3 and 4 present matrices of performance objectives and a selection of the key determinants targeted in the HeLP intervention for each of the three processes of behavior change. The combination of performance objectives, and behavioural determinants, generates (in the cells of the table) the proximal objectives for the Programme (PPOs). These have then been mapped onto the appropriate process of behavior change in the HAPA model. This provided a clear framework to guide the selection and sequencing of the behavior change techniques and practical strategies which constitute the intervention.

### Step 3: Specify behaviour change techniques

The product of step 3 is an inventory of behaviour change techniques selected to match each proximal programme objective. A behaviour change technique (BCT) e.g. 'model/demonstrate behaviour' is a technique designed to change a specified theoretical process or determinant of behaviour. For example, using strategies in the intervention that enable children to practice a targeted behaviour and/or see role models perform the behaviour, is designed to increase self efficacy (confidence in being able to perform the target behaviour), which is a construct of social cognitive theory.

Finding appropriate techniques begins with the question "How can the learning and change objectives (the PPOs) for each performance objective be accomplished?" Methods for identifying suitable techniques included a) discussions with stakeholders, and experts in behaviour change (behavioural science academics/health promotion staff); b) reference to a taxonomy of behavioural change techniques [43,44]; c) consideration of theory and practice in other school-based interventions; d) applying criteria for feasibility, acceptability and cost within a school setting.

A range of suitable BCTs were then selected and included: role modelling, skill and knowledge building, communication skills training, self monitoring, problem solving, modelling/demonstrating behaviour, barrier identification, goal setting, decision balance and social support. For example, to practice skills to communicate the desired healthy lifestyle messages to their parents and seek their support, children modelled and demonstrated the behaviour by participating in a variety of role play scenes, followed up with discussions of issues led by the drama facilitator. Many BCTs may need to be applied to bring about a single PPO e.g. for children to 'practice skills to resist temptation' (PPO number 32, see Table 2), the BCTs used were 'prompt barrier identification', 'problem solving', 'decision balance', 'model/demonstrate behaviour' and communication skills training'. This linked to the PO of 'understand and resist temptation'. (see Table 5).

**Table 5:** Behaviour change techniques and strategies for performance objectives associated with 'Establish Motivation'

Performance objectives	Behaviour change techniques (theoretical framework)	Implementation strategies
**A** Communicate healthy lifestyle messages to parents and seek their help and support	Exchange information (IMB) Prompt barrier identification Model/demonstrate behaviour Communication skills training (SCT) Prompt identification as a role model (SCT)	Children learn about the healthy lifestyle messages and support strategies through a variety of individual and group tasks delivered by the teacher in PSHE lessons and by actors in drama workshops. '80/20' used as a general message throughout suggesting we should eat healthily and be active at least 80% of the time. Parent information sheets given to children following each drama workshop. Characters and children role play scenes to communicate messages to parents and seek their support. Discussion and role play of ways to encourage whole family to make changes. Characters present scenes, where after having made changes to their behaviours, become role models to others (siblings, parents, friends) followed by group discussion.

**B** Select and try healthy alternatives to unhealthy snacks and drinks at home and at school	Exchange information (IMB) Provide encouragement Modelling (SCT)	Children view and discuss with their chosen character ingredients of both healthy and unhealthy food and drink. Compare fat, sugar and salt content to recommended guidelines. Children observe characters taste healthy snacks and drinks while role playing in different settings Characters provide encouragement Children taste healthy snacks and drinks with their chosen character

**C** Select feasible active alternatives to sedentary activities	Modelling (SCT)	Children and actors role play home and school scenes focussing on replacing sedentary leisure pursuits with active alternatives. Children play interactive games to choose and mime active leisure pursuits. Children observe the characters mime their 24 hour clock and discuss their activity in relation to the '80/20' message.

**D** Understand and resist temptation	Prompt barrier identification (SCT) Problem solving (SCT) Decision balance (SCT) Prompt barrier identification (SCT) Model/demonstrate behaviour (SCT) Communication skills training (SCT)	Children make personalised 'Temptation T shirts' Children work with their chosen character to prepare ways to tempt the other 3 characters and help their own character to resist temptation. Children participate in the 'Temptation Ladder' activity that enables them to practise skills to resist temptations and help others. Children observe characters role play marketing scenes Children participate in the role play.

Theoretical framework: IMB = Information Motivation Behavioural Skills Model; SCT = Social Cognitive Theory

### Step 4: specifying practical strategies and designing the intervention

The implementation strategy is simply the process for delivery of a particular behavior change technique. The strategy needs to be appropriate for the target population and the setting in which the intervention will be conducted. We were mindful (as per our needs assessment) that strategies chosen needed to be deliverable by teachers and relevant external groups operating within a school setting, dovetail with healthy lifestyle initiatives already going on in schools at the time and, where possible, meet National Curriculum requirements for this age group.

#### a) Specifying implementation strategies

It was clear the strategies chosen to deliver the key messages needed to inspire and motivate the children so that they discussed the Programme at home with their parents and each other. Previous research has suggested that drama may be an appropriate means of engaging children, increasing knowledge and changing health behaviours [30-33]. Following discussions with experts in education and drama, it was hypothesised that interactive drama based activities where the children take ownership of the issues was more likely to motivate children to become engaged with the process, make changes and to engage their parents than passive receipt of messages. We also hypothesised that, if the children were involved in the development of materials, including the scenarios they produce (facilitated by actors), they would be more likely to be receptive to the health messages. Drama sessions were also compatible with the existing school curriculum and could provide a framework within which to deliver many of the proposed behaviour change techniques in a way which is accessible and engaging for children.

Table 5 presents a summary of behaviour change techniques and implementation strategies chosen to accomplish PPOs (not shown) for each performance objective related to 'establishing motivation'. To view the table showing techniques and strategies for 'take action' and 'stay motivated' see Additional file 2.

#### b) Designing the Programme

Utilising the Health Action Process Model, our chosen implementation strategies were then ordered to create three intervention components (components 2-4) following the sequence of the three broad behaviour change processes in the HAPA model, with an additional component (component 1) designed to create a receptive context within the school (Table 6). The HeLP intervention runs over 3 school terms (Spring and Summer term of year 5 and Autumn term of year 6) so that it is feasible and acceptable to schools and to encourage transfer of knowledge and skills into year 6. Table 6 provides a summary of each component of the intervention (with timescales), summarised implementation strategies and their associated performance objectives. Performance objectives marked * for component 1 have not been discussed in this paper as they do not relate directly to the child but a detailed intervention specification showing performance objectives for all target groups is available to view (See Additional file 1).

**Table 6:** The HeLP Programme and associated POs

Component	Process of Behaviour change	Summary of implementation strategies	Performance objectives (POs)
*Component 1* Engaging schools, children & families Spring term (Yr 5)	Establish motivation and create a receptive environment	Whole school assembly Activity workshops Parents' evening Newsletter articles	*Senior management team (SMT) see that the Programme benefits the school and the children and dovetails with existing school initiatives *Year 5 teachers see that the programme is feasible and acceptable to them and their children and does not substantially increase their workload Methods of delivery enthuse children *Parents understand the value of the Programme

*Component 2* Intensive Healthy Lifestyles Week - one week Summer term (Yr 5)	Establish motivation by developing children's confidence and skills and helping them make decisions	PSHE lessons (morning) §Drama (afternoon) (forum theatre; role play; food tasting, discussions, games etc)	A, B, C, D (see Table 2)

*Component 3* Goal Setting - goals set during week following drama Summer term (Yr 5)	Take action by helping children create an action plan and implement goals.	Questionnaire to enable children to reflect on snacking, consumption of fizzy drinks and physical activity. Goal setting sheet to go home to parents to complete with child. 1:1 goal setting interview Parent's evening (child involvement - Forum Theatre)	E, F (see Table 3)

*Component 4* Reinforcement activities - one term post-intervention Autumn term (Yr 6)	Stay motivated by helping children to monitor, assess and adapt goals	Whole school assembly followed by drama workshop to remind school/children of messages. PSHE lesson to remind children of messages and goals. Children monitor goals on personalised chart Class to deliver assembly about the project to rest of school 1:1 goal supporting interview to discuss facilitators/barriers and to plan new coping strategies.	G, H, I (see Table 4)

§The drama framework includes 4 characters, each represented by one of the actors, whose attributes related to the three overall behavioural objectives. Children choose which of the characters they most resemble then work with that actor to help the character learn to change their behaviour

## Piloting

### a) Methods

The intervention was piloted in two phases and process evaluation methods included semi structured interviews (teachers and parents), focus groups (children), questionnaire responses (parents), documentation of parental and child involvement and observations of intervention delivery. The aim of phase 1 was to ensure that the initial intervention components were feasible, appropriate and suitably engaging for the target population (8-11 year olds). We therefore worked with children, parents and teachers from a single primary school to assess a variety of possible activities, materials and modes of delivery to 119 children from three age groups (8-9 year olds; 9-10 year olds and 10-11 year olds) using education lessons and either drama or goal setting. Based on the results/feedback from phase 1, the intervention was further developed and a second phase of piloting took place in a second primary school, in an area of high deprivation, with 77 children from three year 5 classes (aged 9-10 years). The aim of the second phase was to assess 'proof of concept' (i.e whether the intervention could change obesity related behaviours) and the feasibility of taking measures. Height, weight, waist circumference, % body fat, objective physical activity (using accelerometry), food intake (using an adapted version of the Food Intake Questionnaire) [45] and screen time (using an adapted version of the Childrens' TV Viewing Habits Questionnaire [46] were measured at baseline and 6 weeks post intervention.

### b) Results

#### Pilot 1(one primary school; n = 119 children, aged 8-11)

In the questionnaire feedback, many parents reported positive parent/family behaviour changes. Qualitative data from teachers, children and their parents suggested that Year 5 s (9-10 year olds) were more receptive to the messages than the year 4 and 6 children and more able and willing to translate them into possible behaviour changes. In addition, it appeared that this year group engaged their families to the greatest extent. Teachers thought that the education lessons should be taught consecutively over one week to maintain momentum and that the drama and goal setting had the potential to work synergistically by engaging the children through the drama and following this up with encouraging the children, with their parents support, to make changes through setting simple goals. Parents and children also highlighted the need for a greater variety of activities to introduce the key messages and concepts in order to engage both boys and girls.

##### Implications

In order to build a trusting relationship, a range of activities was developed to introduce the school, children and their families to the project's key messages. A 'Healthy Lifestyles Week' was developed consisting of education lessons in the morning (delivered by teachers) which dovetailed with interactive drama activities in the afternoon (delivered by a local drama group).

#### Pilot 2 (one primary school; n = 77 children, aged 9-10)

Staff were enthusiastic about the Programme, in part because it met the National Curriculum guidelines for Personal Social Health Education (PHSE) and Citizenship, and importantly because they felt it promoted families' engagement with the school. Some teachers felt that the drama had a positive effect on the self esteem of the children, particularly those with additional learning needs. Some teachers suggested further activities for the subsequent term to reinforce the messages and refocus the children and their parents on their goals. Many parents reported that their family had made lifestyle changes and that their child was willing to try new foods. The children enjoyed the drama activities and felt that they could relate to the characters within the drama framework that made them more motivated to set their own goals. Some children reported that they had started going to more after school clubs. Table 7 below presents some supporting quotes

**Table 7:** Provides example quotations supporting a selection of performance objectives for teachers, children and their families

Performance Objective	Illustrative quotes
Year 5 teachers need to see that the programme is feasible and acceptable to them and their children and does not substantially increase their workload	*'I really appreciated you giving me all the lesson plans and resources and that they linked to the National Curriculum. I found them easy to follow''* *'I knew the drama would work well with our children'* *'The parents evening did increase my workload a bit but I thought it was worthwhile'*

Class teachers need to be enthused by the programme and develop their understanding and appreciation of the issues	*'I enjoyed observing the children in the drama sessions as I saw what a great impact it had on my class'* *'The project inspired me as I saw what a positive effect it was having on the children with statements'* *'It was good for us to have to teach the PSHE lessons as this helped me to understand what the project was about' *

The methods of delivery need to enthuse children so that they discuss messages with their parents and are motivated to seek family support to make small and simple lifestyle changes	*'[Name] talked a lot about the project. She loved the Chiefs and dance visit despite not being coordinated!'* *'The project encouraged [Name] to become interested in cooking and preparing food.'* *'[Name] plays an active part in choosing healthy options when we shop'*

Children need to be able to select feasible, active alternatives to sedentary activities	*'Since moving house, [Name] no longer cycles to school but he realised he misses it so he is now going to cycle to school again even though he has further to go now. It has come from him and that it good'* *'[Name] has definitely increased her activity and chooses this option instead of TV'*

Parents/families need to make changes	*'I buy more fruit and veg '* *'We do more activity as a family now'* *'I try to make her packed lunches more healthy and interesting'* *'We will only buy brown or wholemeal bread now'*

Quantitative data from the pre and post intervention behavioural and anthropometric measures showed a significant self reported decrease in the consumption of energy dense snacks (p = 0.001), TV viewing (p = 0.033). Objective measures of physical activity showed a significant decrease in girls' sedentary behaviours (p = 0.03) and a significant increase in girls' moderate to vigorous physical activity (p = 0.001). We note that this is only before and after data and some measures were self report and therefore unreliable, however, these results did provide 'proof of concept'.

##### Implications

An additional component was added to the intervention - 'reinforcement activities' to take place at the beginning of year 6. In addition, minor refinements were made to the education lessons and the drama scripts to enhance delivery and continuity. Table 6 shows the final intervention components, associated processes of change, implementation strategies and POs.

A paper providing more detail of these two piloting phases, including a randomised exploratory trial has been published [47].

The drama/school assembly scripts for the actors and a step by step guide for the drama facilitator have been manualised to enable delivery by a local theatre/drama group. The PSHE lessons (with learning outcomes relating to the National Curriulum) and their associated resources have also been manualised so that class teachers are able to deliver the sessions with minimum preparation (these can be obtained from the corresponding author).

## Discussion

This paper describes the use of Intervention Mapping to develop a school-based intervention to prevent obesity in children. Intervention Mapping was a useful tool to guide us through the process of developing the HeLP intervention, as was Abraham and Michie's taxonomy of BCTs [43] which helped us to select feasible BCTs for use in the HeLP Programme. However, these tools did not provide much guidance on how to organise these many BCTs and their associated delivery methods into a coherent, efficient and appropriately sequenced framework. As a result we took further steps to select techniques and strategies to fit around a process model of behavior change The HAPA model provided a framework to order the implementation techniques into a coherent, multi component intervention (Table 6) that could run over three school terms and would enable the children and their families to make lifestyle changes.

The HAPA model is consistent with a number of theories of behavior change including social cognitive theory and control theory [35]. We selected the HAPA as being the 'closest fit' to the set of theoretical determinants we had identified. However, although the HAPA model was useful in helping to identify a broad strategy for sequencing the delivery of BCTs, it did not provide complete coverage of the theoretical determinants we identified. Additional processes we have incorporated included the need to address cravings for unhealthy snacks (affective processes) (see table 1) and the need to build a receptive context within the school environment, component 1 of the intervention (see table 6). Two further BCTs (not addressed in the taxonomy) [43,44] we used to help create a receptive context within the school were the 'identification of barriers' with teachers and the senior management team to delivering the intervention within school time and 'discussion of possible solutions' to these. We also understood the importance of 'showing empathy' (i.e. understanding the nature school life) when liaising with teachers and the SMT which, we felt, was key in building a trusting relationship - essential in getting the school to 'buy into' the Programme. (See Additional file 1).

The development process was also consistent with the new MRC framework for the development and evaluation of complex interventions [6] which suggests an iterative approach in which an understanding of context is central. The evidence that existing school-based programmes actually prevent obesity is weak [48]. We recognize that there are important social determinants of behaviours related to diet and physical activity which are difficult to address and which will inevitably militate against successful interventions delivered to individuals and within micro-environments such as schools. Nonetheless we hope that the rigorous approach employed to develop the HeLP Programme and its modification through iterative pilot phases will increase the likelihood of both efficacy and effectiveness. It also provides us with the basis for the process evaluation we are conducting alongside the efficacy trials which will help us to explore possible reasons for its success or failure.

*Strength and Limitations: *This is one of the few studies to describe in detail the theoretical basis, intervention techniques and strategies of an intervention for reducing and preventing childhood obesity. Through the use of IM methods, the theoretical basis, behavior change techniques and implementation strategies can be seen to fit together as a coherent intervention model. Each technique delivered has a clear purpose and a clear place in the model. The IM approach was very useful, although there was a tendency to generate a long list of behavior change techniques, which were not necessarily coherent or compatible. We were able to correct this by application (and extension) of a process model of behavior change (The HAPA model). In order to make sure that certain behaviours were targeted (such as *encouraging the children to engage their parents and talk meaningfully about the project's messages*) we decided to promote some PPOs to POs so that there could be a second level of 'peeling down' that would enable us to establish more focused and specific implementation strategies related to a key programme objectives.

*Future directions: *Two stages of piloting and refinement of the intervention have taken place. In addition an exploratory randomised controlled trial has just been completed involving 202 children to establish feasibility and acceptability of the Programme and the trial design for a future large cluster RCT. The results of this pilot work will be reported elsewhere.

*Implications for practice*: Interventions to address overweight/obesity (and other complex behavioural interventions) in children could adopt similar methods to clearly outline their intervention methods and the causal processes hypothesised to underlie the desired changes in child and parent behaviour. We believe that this framework allows a deeper understanding of the processes through which such interventions work, improving our ability to design and deliver consistently effective interventions.

## Conclusion

Although time consuming, we found intervention mapping to be a useful tool for developing a feasible, theory based intervention aimed at motivating children and their families to make small sustainable changes to their eating and activity behaviours. The next phase of the research will involve evaluating the effectiveness and cost effectiveness of the HeLP Programme in a large scale cluster RCT.

## Competing interests

The authors declare that they have no competing interests.

## Authors' contributions

JL and KW led the intervention mapping process and conducted the literature review with CG providing advice on the use of IM and BCTs. JL led the design of intervention delivery methods and the production of intervention materials, coordinated the implementation of the intervention during the piloting phases and conducted interviews with teachers and parents. JL drafted the manuscript and provided the main ideas of this paper with KW, CG and SL providing critical revision. JL and KW conducted the focus groups and with SL designed the study and obtained funding. JL will act as guarantor of the paper. All authors read and approved the final manuscript.

## Supplementary Material

Additional file 1 **Detailed Intervention Specification of the HeLP Programme**. Click here for file

Additional file 2 **Behaviour change techniques and strategies for performance objectives associated with 'Take Action' and 'Stay Motivated'**. Click here for file
