# Supplementary material for: Evidence, theory and context - using intervention mapping to develop a school-based intervention to prevent obesity in children
Source: Int J Behav Nutr Phys Act. 2011 Jul 13;8:73. doi: 10.1186/1479-5868-8-73 (PMC3152876; doi:10.1186/1479-5868-8-73)
Supplement: Additional file 1 — Detailed Intervention Specification of the HeLP Programme. [file 1479-5868-8-73-S1.DOC]

**Additional file 1 - Detailed Intervention Specification of the HeLP Programme**

| **Performance Objective**  (mapped onto appropriate process of behaviour change) | **Target Group** | **Determinants of Performance Objectives (personal – blue; external – black)** | **Behaviour change technique (Theoretical framework)** | **Implementation Strategies** | **Tools** |
| --- | --- | --- | --- | --- | --- |
| **Establishing motivation (Engagement)**  i) Senior management need to see that the Programme benefits the school and the children and dovetails with the existing year 5 curriculum and school initiatives already in operation.  **Performance Objective**  (mapped onto appropriate process of behaviour change) | School senior management team  **Target Group** | Perceived importance (school priorities)  Feasibility and acceptability of the Programme for the school  Self efficacy  **Determinants of performance objectives** | Exchange information (elicit-provide-elicit) based on motivational interviewing  (IMB)  Identify possible barriers and solutions  **Behaviour change technique (Theoretical framework)** | Present the HeLP Programme to primary heads at the regional Association for Primary Heads briefing detailing the rationale for the research study and results of completed pilot work. Year 5 teacher and head teacher on our advisory committee to present their views and experiences of being involved with the Programme. Question and answer session.  Leaflets detailing the project given out to attendees.  Meet with head/member of senior management team at the school to discuss the project further and to reiterate the benefits it can bring to the school  (*Spring/Summer term before baseline measures)*  **Implementation strategies** | Handout summarising the rationale and background to the project.  Project timetable  **Tools** |
| ii) Year 5 teachers need to see that the Programme is feasible and acceptable to them and their children and does not increase their workload | Year 5 class  Teachers | Support available from project team and senior management  Perceived importance  Feasibility/acceptability | Exchange information (IMB)  Showing empathy  Identify barriers and solutions | Meet with year 5 teachers to discuss the Programme (rationale, pilot work completed,  the intervention). Allay any fears concerning increased workload. Discuss the benefits for them and their class (*Summer term before baseline measures)*  Hold a staff meeting to present the project to all staff and to discuss how each year group can utilise their present curriculum to incorporate the HeLP messages into class and homework (e.g. Poem/rap based on the messages in 2 literacy lessons; food tasting, planning a healthy lunch box, watching and analysing food adverts aimed at children, recording physical activity, making personal a 24 hour activity clock and looking at time spent in active and sedentary pursuits) *(Autumn term Yr 5)* | Project timetable |
| **Performance Objective**  (mapped onto appropriate process of behaviour change) | **Target Group** | **Determinants of performance objectives** | **Behaviour change techniques**  **(Theoretical framework)** | **Implementation strategies** | **Tools** |
| iii) The methods of delivery need to enthuse children so that they discuss messages with their parents and are motivated to seek family support to make small and simple lifestyle changes.  **Performance Objective**  (mapped onto appropriate process of behaviour change) | Children  **Target Group** | Learning preferences  Ability to identify with the 4 characters and the drama content  **Determinants of performance objectives** | Focus on fun and enjoyment (TPB – perceived behavioural control leads to greater enjoyment)  Rewards  Use of role models  e.g. health behaviour linkage, risk awareness, positive benefits of healthy lifestyle  Exchange information (IMB)  Problem solving (SCT)  Empowerment (CMT/TPB/SDT)  Use of active learning techniques (problem solving, group interaction, role play, setting homework, performance)  **Behaviour change techniques (Theoretical framework)** | Interactive whole school assembly with the launch of a rap competition based on the project’s key messages (prizes and certificates for winners) *(Spring term Yr 5)*  Delivery of fun and engaging activity workshops with local professional sportsmen/dancers/chefs/vegetable growers *(Spring term Yr 5)*  Specially devised PSHE lessons delivered by the class teacher followed by dynamic interactive drama workshops delivered by young actors. (*Drama framework includes 4 characters whose attributes relate to the healthy lifestyle messages. All characters are likeable and are part of a friendship group. Children choose which*  *of the characters they most resemble and then work closely with that actor during the week to help them change their behaviour).* Encourages ownership of issues  (*Summer term Yr 5)*  **Implementation strategies** | Powerpoint presentation  Competition entries  Sports equipment  Music and stereo system  School hall/playing field, Cooking equipment Gardening equipment  PSHE lessons and associated resources,  School hall,  Actors, drama scripts, facilitator guide  Various props  **Tools** |
| iv) Family needs to understand the value of the Programme for promoting the health and wellbeing of the whole family | Parents/family | Nutrition/PA knowledge and beliefs  Perceived norms  Stage of change | Exchange information on health behaviour link  (IMB)  Raising awareness of family issues (TM)  Prompt identification as a role model (SCT/SET) | Parents’ evening to introduce the parents to the project team and to present the research and its rationale (information about the consequences of unhealthy lifestyles for children;  the complex nature of behaviour change and the importance of whole family involvement; pilot work already untaken and a brief overview of the intervention)  *(Spring term Yr 5)*  Parents invited to observe  drama workshops (particularly forum theatre* where actors act out family scenes with children stopping the action, if inappropriate, suggesting changes to improve outcomes and then acting out their suggested changes within the scene) *(Summer term Yr 5)* | Powerpoint presentation  Paediatrician not Research Fellow to provide information on the health implications  Actors, facilitators, drama script |
| **Establishing motivation (Develop confidence and skills, make decisions)**  i) Children communicate messages to parents and seek their help and support  **Performance Objective**  (mapped onto appropriate process of behaviour change) | Children  **Target Group** | Nutrition/PA knowledge and attitudes  Perceived norms  **Determinants of performance objectives** | Exchange information (IMB)  **Behaviour change techniques (Theoretical framework)** | Children learn about the healthy lifestyle messages through a variety of individual and group tasks delivered by the teacher in the PSHE lessons and by actors in the drama workshops  **Implementation strategies** | 5 specifically designed PSHE lessons plans and associated resources which link closely to each drama interactive workshop.  **Tools** |
| **Performance Objective**  (mapped onto appropriate process of behaviour change) | **Target group** | Confidence/self  efficacy  Norms  Modelling  Reinforcement  Communication skills  **Determinants of performance objectives** | Prompt barrier identification (SCT)  Model/demonstrate behaviour (SCT)  Communication skills training (SCT)  Prompt identification as a role model (SCT)  **Behaviour change techniques (Theoretical framework)** | Children take home parent information sheets each day after the drama workshops to encourage discussion  Children take home homework that promotes discussion between child and family members  Characters and children role play scenes in which the characters and children communicate messages to their parents and brainstorm ways to encourage the whole family to become involved in making lifestyle changes. Followed by group discussion  Characters role play scenes where, after having made changes to their behaviours, become role models to  others (e.g. siblings, parents, friends)  Followed by group discussion  (*Summer term Yr 5)*  **Implementation strategies** | 5 parent information sheets(*traffic light food system, breakfast, snacking, lunch boxes, strategies)*  Homework sheets  Actors, facilitator, scripts, props  **Tools** |
| ii) Children need to be able to select and try healthy alternatives to unhealthy snacks and drinks. | Children | Nutrition knowledge  Confidence/self efficacy  Food preferences  Perception of taste  Familiarity of foods  Perceived norms | Exchange information  (IMB)  Active learning  Provide encouragement  (SET)  Modelling (SCT) | Children look at and discuss with their character ingredients of both healthy and unhealthy food and drinks. They then compare their fat, sugar and salt content to recommended guidelines.  With their characters, children are given a variety of healthy snacks and drinks to taste after having first observed the characters role play tasting the foods themselves and receiving encouragement and praise from the other characters (role play a variety of settings)  *(Summer term Yr 5)* | A selection of laminated food packages and their ingredients, the Which Shoppers Food Guide card and actors  Healthy snacks/drinks (fruit, nuts, seeds, dips, bread sticks, oat cakes, cereal bars, no sugar squash and fizzy water and smoothies). Actors and facilitator |
| **Performance Objective**  (mapped onto appropriate process of behaviour change) | **Target group** | **Determinants of performance objectives** | **Behaviour change techniques (Theoretical framework)** | **Implementation strategies** | **Tools** |
| iii)Children need to be able to select feasible active alternatives to sedentary activities | Children | Physical activity knowledge | Modelling (SCT) | Children and actors role play scenes and play a variety of interactive games to choose and mime alternatives to sedentary behaviours.  Children watch characters mime their 24 hour activity clock and then these are discussed with the facilitator in relation to the 80/20 message (*Summer term Yr 5)* | Actors, facilitator and drama script and associated props |
| iv) Children need to be aware of what tempts them to make unhealthy choices and be able to resist temptation with the support of their parents and friends  **Performance Objective**  (mapped onto appropriate process of behaviour change) | Children  **Target group** | Self evaluation  Confidence/self efficacy  **Determinants of performance objectives** | Prompt barrier identification (SCT)  Problem solving (SCT)  Decision balance  Modelling (SCT)  Prompt barrier identification (SCT)  **Behaviour change techniques (Theoretical framework)** | Children make personalised ‘Temptation T shirts’ in a PHSE lesson after having collected wrappers and pictures of foods/activities that tempt them to make unhealthy choices  In the drama workshop children wear their T shirts and work with their characters to prepare ways of tempting one of the other characters to make unhealthy choices. They also prepare ways to help their own characters resist temptation.  Each character then steps up to the ‘Temptation Ladder’ and one child from another group tries to tempt them with an unhealthy food or  **Implementation strategies** | Old T shirt and unhealthy food wrappers and pictures of sedentary activities  Actors, facilitator, drama script  Blue and red hoops for the ‘temptation ladder’, actors, facilitator and drama script  Temptation T shirts  **Tools** |
|  |  |  | Model/demonstrate behaviour (SET)  Communication skills training (SCT) | activity. The character is allowed to have a helper from their group to be a voice in their ear helping them resist. If the character makes the decision to resist he/she steps into the blue hoop, but if the character gives in to temptation they step into the red hoop. If they are undecided they put one foot into the red hoop and one into the blue hoop. This activity continues with different children helping/tempting until the character has reached the top of the ‘hoop ladder’. Once all the characters have.  completed the ladder the children then have ago themselves at resisting temptation  Children role play marketing scenes  (*Summer term Yr 5)* | Actors, facilitator, drama script |
| **Performance Objective**  (mapped onto appropriate process of behaviour change) | **Target group** | **Determinants of performance objectives** | **Behaviour change techniques (Theoretical framework)** | **Implementation strategies** | **Tools** |
| v) Class teachers need to be enthused by the Programme and develop their understanding and appreciation of the issues | Class teachers | Nutrition/PA knowledge and beliefs  Self efficacy | Exchange information and ideas for working with children in an interactive and dynamic way (IMB/SCT/CMT) | Teachers observe all the drama workshops during the Healthy Lifestyles Week  *(Summer term Yr 5)* |  |
| **Take action (create an action plan and implement it)**  i) Children need to reflect upon their own snacking and leisure choices  **Performance Objective**  (mapped onto appropriate process of behaviour change) | Children  **Target group** | Self evaluation  Norms  Modelling  Reinforcement  **Determinants of performance objectives** | Raising awareness (TM)  Prompt intention formation (TPB/SCT/IMB)  **Behaviour change techniques (Theoretical framework)** | During the PSHE lessons children reflect upon their snacking and physical activity habits using individual and group classroom tasks as well completing specific homework tasks  Children complete step 1 (self reflection) of the ‘goal setting sheet’ during the final PSHE lesson  *(Summer term Yr 5)*  **Implementation strategies** | Lesson plans and worksheets  Goal setting sheet  **Tools** |
| ii) Children need to set goals and make changes | Children |  | Implementation  intentions (TPB)  Prompt social support from family (social support theories)  Prompt specific goal setting (CT)  Teach to use prompt or cues (OC)  Coping plan | Child completes step 2 of the ‘goal setting sheet’ (setting goals) with parents. (child sets 3 SMART goals indicating *what* they will replace (e.g. TV), *when* they will replace it and 3 feasible alternatives).  For each goal set with their parents (see below) children write what strategies they can employ to help with goal achievement.  In the presence of their parents, children write down the support they need to achieve their goals (Step 3 of the ‘goal setting sheet’)  Children have a 1-1 discussion about agreed goals with a researcher. Look at main barrier for achieving each goal and discuss and record a coping strategy for each  *(Summer term yr 5)* | Goal setting sheet  Goals and action plan sheet  Pedometers (given as a motivational tool) |
| iii) Parents need to reflect upon their child’s and their family’s eating and activity behaviours  **Performance Objective**  (mapped onto appropriate process of behaviour change) | Parent(s)  **Target group** | Nutrition/PA knowledge and skills  Stage of change  Perceived norms  **Determinants of performance objectives** | Raising awareness (TM)  Prompt specific goal setting (CT)  **Behaviour change techniques (Theoretical framework)** | Parent(s) helps child complete  Steps 2 and 3 of the ‘goal setting sheet’ Parents are also encouraged to write down *how* they/the family will help the child achieve their goals e.g. *offer fruit instead of*  **Implementation strategies** | Goal setting sheet  **Tools** |
|  |  |  | Implementation intentions (TPB)  Prompt social support from family and child  (social support theories)  Agree on behavioural contract (OC) | *biscuits as a snack*  In the presence of their child, parents write down the support they need to help their child achieve goals (Step 3 of the Goal Setting Sheet) e.g. *Child to help shop for healthy snacks they enjoy*  Child and parent sign and date the ‘Goal Setting Sheet’  Parents, child and class teacher receive copy of the agreed goals  *(Summer term yr 5)* | Goals sheet  Fridge magnet  Letter for parents  Post it notes |
| iv) Parents/families need to make changes | Parents | Stage of change  Motivation  Confidence/self efficacy  Nutrition/PA knowledge and beliefs | Prompt identification as a role model (SCT/SET) | Parents’ evening showing drama scenes and using forum *theatre (involvement of children in scenes with the actors to show the importance of social support and parents as role models in helping characters achieve goals).  *(Summer term yr 5)* | Actors, facilitator, props |
| **Stay motivated (monitoring progress, assess and adapt goals)**  i) Children need to monitor their behaviours | Children | Self evaluation  Norms  Modelling  Reinforcement | Prompt self monitoring of goals (CT) | In class children produce a personalised self monitoring chart  Copy sent home and a copy kept at school (Summer term Yr 5)  Every 3 weeks children look at and record progress on their personal chart *(Autumn term Yr 6)* | Card, paper, pens, stickers |
| **Performance Objective**  (mapped onto appropriate process of behaviour change) | **Target group** | **Determinants of performance objectives** | **Behaviour change techniques (Theoretical framework)** | **Implementation strategies** | **Tools** |
| ii) Parents need to monitor their child’s behaviours | Parents | Stage of change  Norms  Modelling  Reinforcement | Prompt monitoring of family/child goals (CT) | Children take home self monitoring chart with a letter for parents (importance of monitoring goals and family support)  (*Summer term Yr 5)* | Letter to parents |
| iii) Children need to assess barriers to goal achievement | Children | Norms  Modelling  Reinforcement  Self efficacy  Self evaluation | Prompt review of behavioural goals (CT)  Prompt barrier identification (SCT)  Coping plan | Drama workshop delivered by the actors to include role play of barriers the children have experienced  PSHE lesson delivered by class teacher to look at barriers and facilitators to goal achievement  1-1 goal supporting interview with researcher to discuss facilitators and barriers to goal achievement and plan a new coping strategy to aid goal achievement  *(Autumn term Yr 6)* | Actors, facilitator and props  Teacher, lesson plan and associated resources  Copy of goals from Yr 5  Goals/Coping recording sheet |
| iv) Children need to adapt goals | Children | Nutrition/PA knowledge  Self efficacy | Prompt intention formation(TPB,SCT,IMB) | Children agree adapted goals with researcher during 1-1 discussion *(Autumn term Yr 6)* | Goals written on goals/coping record sheet and sent home for parents to sign. |
| v) Teachers need to reinforce behaviour change | Class teacher | Perceived importance  Self efficacy | Prompt review of behavioural goals (CT) | Whole class behaviour chart in which children can indicate any changes they have made using stickers *(Autumn term Yr 6)* | A3 laminated class behaviour chart and stickers |

**Theories**

IMB = information motivation behavioural skills model; TPB = theory of planned behaviour; SCT/ SET = social cognitive theory/self efficacy theory

CT = control theory; CMT = competence motivation theory; TM = transtheoretical model; OC = operant conditioning; SDT = self determination theory

**Forum Theatre** ***Forum Theatre** is a type of [theatre](http://en.wikipedia.org/wiki/Theatre) whereby audience members can stop a performance and suggest different actions for the actors to carry out on-stage in an attempt to change the outcome of what they are seeing. This method brings audience members into the performance enabling them to have an input into the dramatic action they are watching. In the HeLP Programme, this method is used so that the children have the power to change and participate in scenes in which the actors show unhealthy/negative behaviours.
