# Supplementary material for: Evidence, theory and context - using intervention mapping to develop a school-based intervention to prevent obesity in children
Source: Int J Behav Nutr Phys Act. 2011 Jul 13;8:73. doi: 10.1186/1479-5868-8-73 (PMC3152876; doi:10.1186/1479-5868-8-73)
Supplement: Additional file 2 — Behaviour change techniques and strategies for performance objectives associated with 'Take Action' and 'Stay Motivated'. [file 1479-5868-8-73-S2.DOCX]

Additional file 2 – Behaviour change techniques and strategies for performance objectives associated with ‘Take Action’ and ‘Stay Motivated’

| **Performance objectives** | **Behaviour change techniques**  **(theoretical framework)** | **Implementation strategies** |
| --- | --- | --- |
| **E** *(Take Action)*  Reflect on own snacking and leisure choices | Raising awareness (TM)  Prompt intention formation (TPB, SCT, IMB) | Children reflect on snacking and leisure choices in individual and group classroom tasks as well as homework tasks  Children complete step 1 (self reflection) of the ‘goal setting sheet’ |
| **F** *(Take Action)*  Set goals and make changes | Implementation intentions (TPB)  Prompt social support from family (Social Support theories)  Prompt specific goal setting (CT)  Teach to use prompts or cues (OC)  Coping plan  Model/demonstrate behaviour  Prompt identification as a role model (SCT) | For each goal (set with parents) children write what strategies they can use to help with goal achievement  In presence of parents, children write down the support they need to achieve their goals (step 3 of ‘goal setting sheet’)  Children have 1-1 discussion about agreed goals, possible barriers and coping strategies with researchers (goals and strategies sent directly home to parents)  Children given pedometer as a motivational tool  Children participate in Forum Theatre*  Parents observe/participate in Forum Theatre |
| **G** *(Stay Motivated)*  Monitor goals | Prompt self monitoring of goals (CT) | Children produce a personalised self monitoring chart in class (taken home with letter to parents). A copy of this chart is kept in school for children to look at and complete every three weeks |
| **H** *(Stay Motivated***)**  Assess barriers to goal achievement | Prompt review of behavioural goals (CT)  Prompt barrier identification (SCT)  Coping plan | Group and individual class activities to assess facilitators and barriers to goal achievement  Children observe and participate in scenes with characters to role play barriers experienced  1-1 goal supporting interview with researcher to discuss facilitators/barriers to goal achievement and plan new coping strategies to aid goal achievement |
| **I** *(Stay Motivated)*  Adapt goals | Prompt intention formation (TPB, SCT, IMB) | Children agree adapted goals with researcher and parents (new goals and strategies sent home to parents) |

***Forum Theatre** is a type of [theatre](http://en.wikipedia.org/wiki/Theatre) whereby audience members can stop a performance and suggest different actions for the actors to carry out on stage in an attempt to change the outcome of what they are seeing. This method brings audience members into the performance enabling them to have an input into the dramatic action they are watching. In the HeLP Programme this method is used so that the children have the power to change and participate in scenes in which the actors show unhealthy/negative behaviours.

Theoretical framework - IMB = information motivation behavioural skills model; TPB = theory of planned behaviour; SCT = social cognitive theory; CT = control theory; CMT = competence motivation theory; TM = transtheoretical model; OC = operant conditioning; SDT = self determination theory
